# Using coronary sinus ostium as the reference for the slow pathway ablation of atrioventricular nodal reentrant tachycardia in children

**DOI:** 10.1002/joa3.12379

**Published:** 2020-06-11

**Authors:** Ming‐Lon Young, Jianli Niu

**Affiliations:** ^1^ Heart Institute Joe DiMaggio Children’s Hospital, Memorial Healthcare System Hollywood FL USA; ^2^ Office of Human Research Memorial Healthcare System Hollywood FL USA

**Keywords:** atrioventricular nodal reentrant tachycardia, catheter ablation, cryoablation, radiofrequency ablation, triangle of Koch

## Abstract

**Background:**

Successful slow pathway (SP) ablation sites for atrioventricular nodal reentrant tachycardia (AVNRT) are usually located inside the Koch's triangle (KT). This study aimed to determine the ablation site of SP using the coronary sinus (CS) ostium (CSO) as the reference and to evaluate the efficacy of the CSO‐guided SP ablation.

**Methods:**

A regional geometry around the KT was constructed by 3D mapping in 52 consecutive patients under age 18 with AVNRT. SP cryoablation was performed. If initial cryoablation was unsuccessful or cryoablation was deemed not suitable, then radiofrequency (RF) ablation was performed. The successful ablation site direction relative to the CSO was expressed as o'clock with the CSO viewed as a clock.

**RESULTS:**

Cryoablation was used as the primary energy source in 40 patients. Of which, 32 were successful and eight required additional RF ablation. Direct RF ablation was performed in 11 patients. Using the CSO as reference, the successful site with cryoablation was at its 2.2 ± 0.6 o'clock; the RF ablation success site was at CSO 2.7 ± 0.5 o'clock (*P* = .006). During a median follow‐up of 12 month, there was 98% success of SP ablation in these patients, with one patient with RF ablation had a tachycardia recurrence.

**Conclusions:**

Using CSO as reference, the cryoablation site at its 2:00 o'clock and RF ablation at its 3:00 o'clock are highly efficacious for SP ablation with good short‐term outcomes, and may be a useful tool in guiding the ablation target for AVNRT.

## Graphical abstract

Ablation success sites in cryoablation group and RF ablation group: Using CS ostium as reference and viewed as a clock, the median (solid line) and mean (dashed line) directional location of the successful cryoablation group (green) and RF ablation group (blue) are depicted in an illustrated Koch's triangle in a right anterior oblique view.

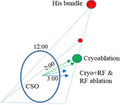

## 1 INTRODUCTION

Catheter ablation of the atrioventricular nodal slow pathway (SP) is widely used for the treatment of atrioventricular nodal reentrant tachycardia (AVNRT) in the pediatric patients. Successful SP ablation sites in the majority of AVNRT cases are located at the lower third of the Koch's triangle (KT) at its posteroseptal region anterior to the coronary sinus (CS) ostium (CSO) between the CSO and tricuspid annulus.^1^ Although the size of the CS and the distance between the His bundle (HB) and CS are proportional to body weight, height, and surface area in children,^2^ the geometry of KT and the CSO have a wide variation.^3^ However, nowadays by using 3D electroanatomic mapping system we can accurately depict KT in each individual patient to overcome the variation and be more effective in SP ablation than using the traditional fluoroscopic method.^4^ Also, the successful sites of SP ablation can be precisely documented by using the individually reconstructed CSO as the location reference.

The energy source for SP ablation can be either by cryothermal (Cryo) or radiofrequency (RF) energy. Because RF ablation is associated with a small and not negligible risk of irreversible complete heart block, cryoablation has emerged as the primary energy source in many centers,^5^ and RF energy is being used as a backup if cryoablation is deemed unsuccessful. Although cryoablation is safer, to use this energy modality it requires reliable testing protocol to elicit SP or patients in stable AVNRT during cryo mapping period to identify the potentially successful ablation site before proceeding to a full‐length ablation. In patients lacking these parameters, RF energy is used as the primary energy source and search for the site where ablation induces accelerated junctional beats where SP ablation can be successfully achieved. Herein, we report our experience with use of the 3D electroanatomic mapping reconstructed CSO as a reference to localize the site for successful SP ablation in patients with cryoablation and/or RF ablation. The primary goal for the study was success rates of SP ablation and sites for cryoablation and/or RF ablation. The secondary goal of this study was to assess the efficacy, safety, and long‐term outcome of our ablation methods.

## 2 METHODS

### 2.1 Study patients

This is a retrospective study from Memorial Regional Hospital of the Memorial Healthcare System in Hollywood, Florida, USA. Medical records of patients under age 18 with structurally normal heart and with the diagnosis of AVNRT (both common and uncommon forms) who had catheter ablation of their SP from February, 2015 to January 2019 were reviewed. Antiarrhythmic medications were discontinued for at least 5 half‐lives before the procedures. Written informed consent was obtained from their parents or legal guardian. Data for patient demographics (age, gender, height, and body mass index), indications for catheter ablation, procedural details, and outcome data (initial and long‐term successful ablation rate, procedural time, fluoroscopic time, and ablation complications) were collected and entered in the database. The protocol for this research project has been approved by the Memorial Healthcare System's institutional review board (Approval No. MHS2018.024; Date of Approval, 06/12/2019) and it conforms to the provisions of the Declaration of Helsinki.

### 2.2 Electrophysiologic study and ablation

Under general anesthesia or deep sedation, femoral venous accesses were obtained. A deflectable 6‐Fr decapolar electrode catheter was advanced from femoral vein into inferior vena cava, right atrium, superior vena cava, and CS under the guidance of the St. Jude Medical NAVX^®^ or Precision^®^ 3‐D electroanatomic mapping system (St. Jude Medical). Respiratory compensation was used in each case during mapping. A precurved long sheath (SR0^®^, St. Jude Medical, Inc) was used in conjunction with an ablation catheter for mapping and ablation. By advancing, withdrawing and deflecting this decapolar catheter in the CS, its ostium was reconstructed. The individual geometries were then combined to form a regional geometry. The right atrial, right ventricular, and HB catheters were then placed. We consistently used 5F hexapolar HB catheter with an interelectrode distance of 2‐5‐2 mm. All HB signals were tagged. The lowest HB recording site, the septal tricuspid valve annulus (delineated at where atrial and ventricular electrograms were simultaneously recorded), and the posterior margin of the CSO (as surrogate of tendon of Todaro) were used to define the triangle of Koch. The SP was initially targeted at the lower third of KT at its posteroseptal region anterior to the CS ostium with local electrogram A/V ratio of about 1:2. We were not actively seeking SP potential (ie, a low‐amplitude activity with a slow rate of rise between atrial and ventricular electrograms, or a low‐amplitude and ‐frequency component followed by a high‐amplitude and ‐frequency component)^6^, ^7^ to guide our ablation during the procedure.

The diagnosis of AVNRT was verified with several electrophysiological criteria, including (a) inducible AVNRT without or with isoproterenol challenge, (b) an atrial‐His (AH) jump diagnostic of dual AV nodal physiology (defined as a sudden prolongation of the AH interval of ≥50 ms when shortening the cycle length of atrial pacing or the coupling interval of the atrial extra stimulus by 10 ms), and (c) patients with clinical narrow QRS tachycardia without inducible AVNRT who had inducible 1 or 2 AVN echo beats, or with sustained PR ≥ RR during atrial overdrive pacing with 1:1 AV conduction prior to AV block, who had no evidence of accessory pathway conduction.

If the patient had reliable test pacing protocol to elicit SP, or had stable AVNRT on baseline study, then we used cryoablation (Freezor Xtra^®^ 6‐mm‐tip catheter, medium curve) as our primary energy source. The catheter tip temperature was set at −80°C for ablation. During sinus rhythm, when the temperature dropped to <−30°C a preselected pacing protocol was started (cryo mapping technique) to assess whether the SP or AV nodal echo beat could be eliminated within 25 seconds. If the patient had no reliable test pacing protocol but had a stable AVNRT in baseline condition (not on isoproterenol), then cryo mapping was performed during AVNRT to see whether the tachycardia could be terminated within 25 seconds at the selected site. If the SP was still present or AVNRT persisted during cryo mapping, the ablation process was aborted and the catheter was moved to a different site. If the cryo mapping was deemed success, cryoablation was proceeded for 4 minutes, followed by two additional ablation of 4 minutes each in a freeze‐thaw‐freeze cycle delivered to the same site. During freeze‐thaw‐freeze cycle, we tried to minimize any unintentional movement of the catheter. If there was unintentional catheter movement, the highest cryoablation point was taken as the success site. Ablation was promptly stopped if the PR interval was prolonged.

If cryoablation could not eliminate the AVNRT either without or with isoproterenol challenge, or the SP persisted with ≥50 ms window or with multiple echo beats, then ablation energy was changed to RF. If there was modification of the SP properties on cryoablation, but then recovery after the freeze, this was also deemed failed lesion. Since this was a retrospective study, there was no preset number of failed lesions before moving to RF energy. In patients with inducible AVNRT but without reliable test pacing protocol to elicit SP, or had the AVNRT was nonsustained on baseline study, then we used RF as our primary ablation energy source.

RF ablation was performed during sinus rhythm with energy setting of 40‐50 Watts and temperature setting of 60°C. If in 15 seconds no junctional beats were observed, the catheter was moved to a new site. If the RF ablation attempt induced accelerated junctional beats, then ablation was continued for 1 minute at this site with atrial pacing if needed to override the ablation‐induced accelerated junctional beats and to monitor the PR interval, followed by 1‐2 additional 1‐minute insurance ablation.

The tag points for both Cryo and RF ablations were all made right before energy application, and monitored for stability throughout. The number of ablation lesions were counted with ablation ≥25 seconds for cryoablation and >15 seconds for RF ablation. The total ablation lesion time in each case was calculated with all cryoablation lesions longer ≥30 seconds and RF ablation ≥15 seconds. Respiration compensation was always performed before model creation. Field scaling was always applied before taking measurements and no sensor‐enabled catheters were utilized. There was no breath holding practice during ablation application.

**FIGURE 1:**
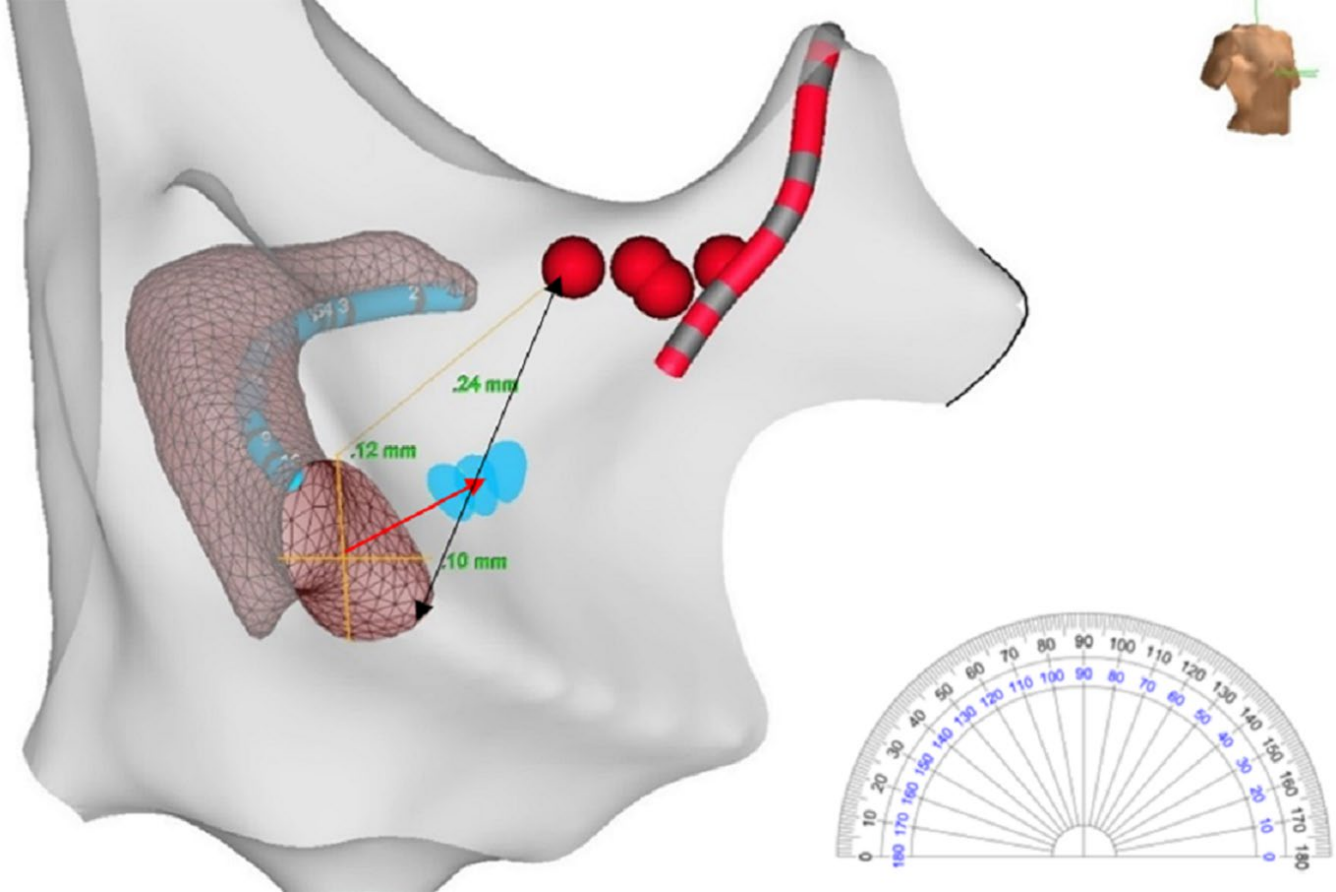
Electroanatomic mapping and SP cryoablation in a patient with AVNRT showing a right anterior oblique view of the right atrium and the CSO. Red balls, HB cloud; Light blue dots, successful ablation sites. Measurements: CSO height and width (12 × 10 mm); from the lowest recorded HB recording to the CSO 12 o'clock site (His‐CS12): 24 mm. The success ablation site vertical location is 37% (counting from the CSO floor; ie, between the middle and lower two third of the KT) of the total length from the HB to CSO floor (black double arrow line passing through the ablation site). A transparent protractor (seen at the side) is aligned to the center of CS ostium to obtain the direction (red arrow) of the success ablation site. In this case, it is at 30° or 2:00 o'clock direction (red arrow) with CSO viewed as a clock. SP, slow pathway; AVNRT, atrioventricular nodal reentrant tachycardia; CSO, coronary sinus ostium. HB, His bundle

The angle of right anterior oblique (RAO) view (around 30‐45°) was decided by best visualizing the cross‐sectional view of the CSO (actually slight difference in the degree will not affect the directional angle of the ablation site (ie, 2 o'clock direction will still be 2 o'clock direction either viewed at 30 or 45° angle). Without tilting of the 3 D image and with a cutting plane allowing the visualization of the interatrial septum and the KT and with the CSO facing the researcher, the distance between the lowest recorded HB site to the CSO 12 o'clock site (the most superior portion of the CS ostium in its superior‐inferior axis: His‐CS12), and the height (from the top edge to the bottom edge of the CS ostium) and width of the CSO, were measured on the 3D reconstructed image. As the shape of the CSO may not be a perfect circle or ellipse, the middle point of height of the CSO was set as the center of the ostium. The ablation site directional location relative to the CSO was obtained by using a transparent online protractor (https://www.ginifab.com/feeds/angle_measurement/) in an uploaded RAO image, and expressed as a directional angle using the CSO viewed as an o'clock. The angle of 0° corresponded to the 3 o'clock position, the angle of 30° corresponded to the 2 o'clock position, the angle of 60° corresponded to the 1 o'clock position, and the angle of 90° corresponded to the 12 o'clock position, respectively. The vertical location was expressed as % of the HB to CSO floor distance (vertical height of the KT) counting from the CSO floor, calculated by drawing a line from the HB to CSO floor passing near the ablation site (Figure 1).

### 2.3 Outcomes and follow‐up

Patients were monitored overnight postablation before discharge, and were followed‐up at the outpatient clinic. They were enrolled in either Multicenter Pediatric and Congenital EP Quality Initiative (MAP‐IT) or in the national Pediatric Electrophysiological Study (Impact) registry for quality control purpose and received telephone follow‐up by the electrophysiological coordinator regularly.^8^, ^9^ Acute SP ablation success rates were defined as absence of inducible AVNRT and no residual SP, or with residual SP but causing ≤1 AV nodal echo beat, without or with isoproterenol challenge. Late success was defined as lack of symptoms, signs, or ambulatory recordings consistent with tachycardia recurrence.

### 2.4 Statistical analysis

Data are expressed as mean ± standard deviation (SD) and median with interquartile range (IQR). Categorical variables are presented as the absolute number of patients and percentage of their group. Comparison of continuous variables was performed using unpaired Student's *t* test, whereas comparison of categorical variables was performed using Chi‐square tests. Kolmogorov‐Smirnov test is used to analyze the distribution between groups. Correlation coefficient between vertical location using KT height as reference and directional location using CSO as reference was performed using a Pearson's test. All tests were two tailed, and a *P* value of <.05 was considered statistically significant. Statistical analysis was performed using GraphPad Prism 7.0^®^ (GraphPad Software, Inc).

## 3 RESULTS

During the study period, a total of 52 consecutive pediatric patients had SP ablation for their AVNRT. Patient demographics and procedural characteristics for AVNRT are summarized in Table 1. The mean age and body weight were 13.7 ± 3.5 years and 55 ± 21 kg, respectively; 39% were male. All patients had structurally normal hearts. One patient had a previous RF ablation of the SP with recurrence of the AVNRT. One patient had both left side accessory pathway and AVNRT ablation and one patient had both ectopic atrial tachycardia and AVNRT RF ablation in the same session. In one patient both cryo and RF ablation at right posteroseptal, midseptal, and proximal CS sites and RF ablation of the left posteroseptal AV nodal extension site (via a transseptal approach) failed. In this patient cryoablation at the right anteroseptal site 5 mm below the His bundle recording during AVNRT terminated the tachycardia in 20 seconds. Ablation was stopped at 32 seconds when the PR interval was prolonged. Cryoablation was performed for 4 minutes at a slightly lower site that did not prolong the PR which resulted in the final success. No additional insurance lesion was performed. This patient was excluded from the analysis.

**TABLE 1:** Demographic and ablation procedural characteristics

	Cryo (n = 32)	Cryo + RF (n = 19)	*P* value
Age, y	13 ± 3.7	14 ± 3.5	.340
Male, n (%)	13 (41%)	7 (37%)	.789
Weight, kg	54 ± 21	56 ± 21	.420
Procedure time, min	153 ± 55	165 ± 62	.491
Fluoroscopic time, min	0.97 ± 1.7	1.6 ± 4.2	.539
Unsuccessful cryoablation attempts	3.6 ± 4.9	17 ± 14	.014
Unsuccessful RF ablation attempts		0.4 ± 0.8	
Cryoablation lesions	5.2 ± 3.8	6.6 ± 4.4	.330
Total cryoablation time, min	20 ± 13	22 ± 16	.320
RF ablation lesions		2.7 ± 2.1	
Total RF ablation time, min		2.3 ± 1.3	
Postablation SP, n (%)	3 (9.3%)	1 (5.2%)	.597
Postablation AVNR, n (%)	5 (16%)	6 (31%)	.181
Complications			
Transient WP, n (%)	4(13%)	3 (16%)	.741
Follow‐up, month	13 ± 10	16 ± 12	.356
Recurrence, n (%)	0	1 (5%)	.190

Note

Data given as mean ± standard deviation (SD) or n (%).

Abbreviations: AVNR, AV node reentry; RF, radiofrequency; SP, slow pathway; WP, Wenckebach periodicity.

Cryoablation was used as the primary energy source in 40 patients. Of which, 32 (80%) were successful (Group 1) and eight required additional RF ablation to achieve success. RF ablation was used as the primary energy source in 11 patients and all had ablation success. Since there was no significant difference for the success sites between Cryo + RF and RF only patients, these 19 patients (Group 2) were combined to form RF ablation group. There was no significant difference between these two groups in their age, gender, or weight distribution (Table 1). As expected, unsuccessful cryoablation attempts in Group 1 patients was significantly lower than the eight patients in Group 2 with failed cryoablation (3.6 ± 4.9 vs 17 ± 14, *P* = .014). The number of unsuccessful cryoablation attempts in Group 1 was significantly higher than the unsuccessful RF ablation attempts in Group 2 patients (3.6 ± 4.9 vs 0.4 ± 0.8, *P* = .0005). There was no significant difference in number of cryolesions and total cryoablation time in Group 1 patients and the eight patients in Group 2 who had unsuccessful cryoablation.

**FIGURE 2:**
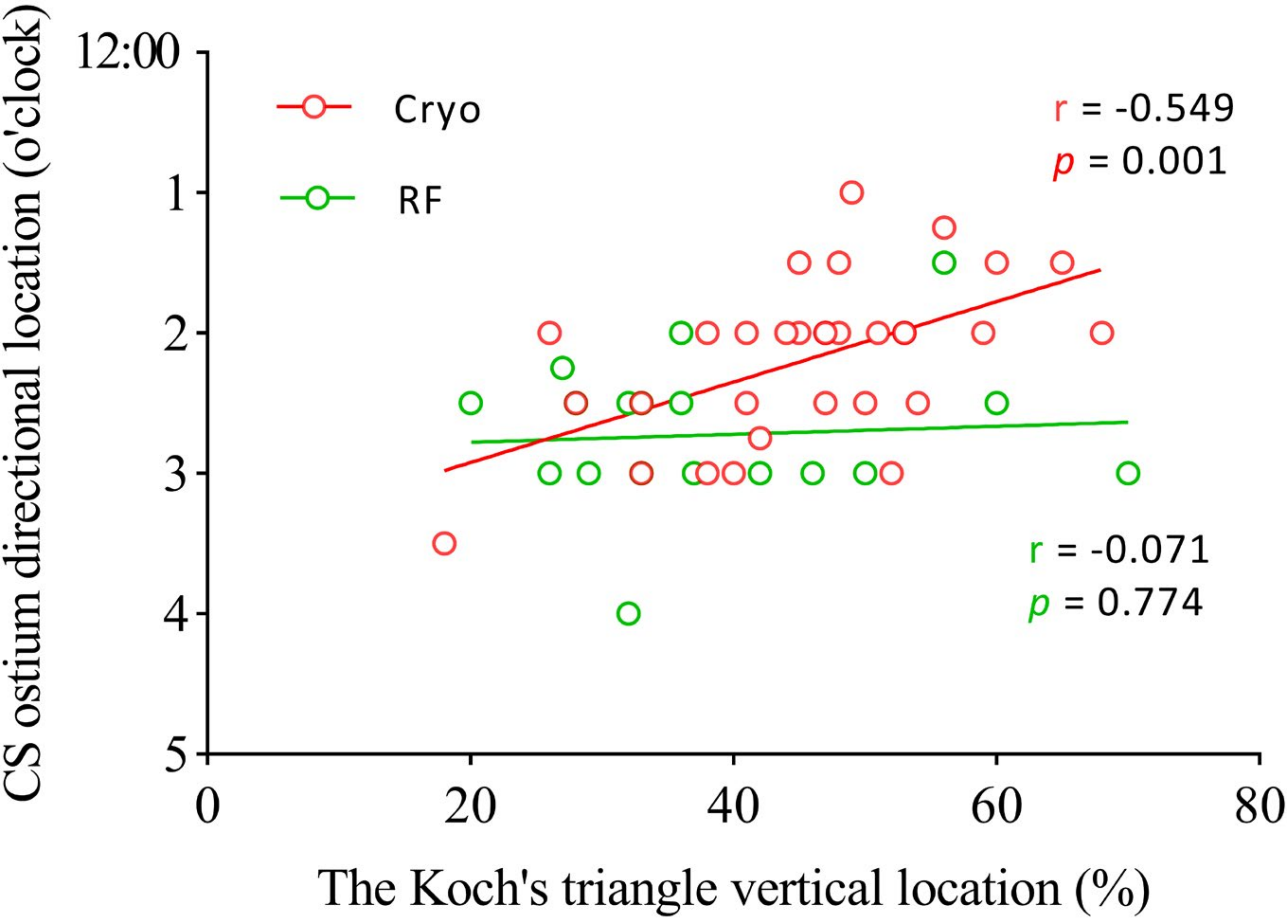
Scatter plots and correlation coefficients for the vertical location vs directional location of both groups. A correlation for the vertical location with the directional location was noted in the cryoablation group (*r* = −.549, *P* = .001), but not for the RF ablation group (*r* = −.071, *P* = .774). RF, radiofrequency; CSO, coronary sinus ostium

Table 2 lists the CSO dimensions, His‐roof of the CS (CS12) distance, as well as the ablation directional location (using CSO as reference and seen as a clock) and ablation vertical location (using the KT height as reference) of these two groups. There was no significant difference in CSO dimensions or His‐CS12 distance in these two groups. Using CSO as the reference, the success ablation site direction for Group 1 (2.2 ± 0.6 o'clock; median 2; IQR: 0.5) was significantly higher than that of Group 2 (2.7 ± 0.5 o'clock; median 3; IQR: 0.1; *P* = .006). Using KT height as reference, the vertical location in Group 1 (46 ± 11%; median 47%; IQR: 13%) was also significantly higher than that of Group 2 (39 ± 13%; median 36%; IQR: 17%; *P* = .009). The correlation coefficient studies of vertical location using KT height as reference vs directional location using CSO as reference of both groups showed that there was a moderate correlation of the vertical location with the directional location for the cryoablation group (ie, high vertical location correlated with high directional location) but not for RF ablation group (Figure 2).

**TABLE 2:** The Koch's triangle dimensions and ablation location

	Cryo (n = 32)	Cryo + RF (n = 19)	*P* value
His‐CS12 distance, mm			
Mean ± SD	22 ± 8.3	21 ± 7.3	.656
Median	21	22	
CSO			
Height, mm			
Mean ± SD	15 ± 3.7	15 ± 3.7	1.000
Median	15.5	15	
Width, mm			
Mean ± SD	13 ± 4.6	12 ± 3.4	.379
Median	12	11	
Directional location (o'clock) using CSO as reference			
Mean ± SD	2.2 ± 0.6	2.7 ± 0.5	.006
Median	2	3	
IQR	0.5	0.1	
Vertical location as % of vertical height of the KT			
Mean ± SD, %	46 ± 11	39 ± 13	.009
Median, %	47	36	
IQR, %	13	17	

Note

Data given as mean ± standard deviation (SD).

Abbreviations: CSO, coronary sinus ostium; CS12, coronary sinus ostium 12:00 o'clock site; KT, Koch's triangle; His, the lowest His bundle recording site; IQR, interquartile range.

**FIGURE 3:**
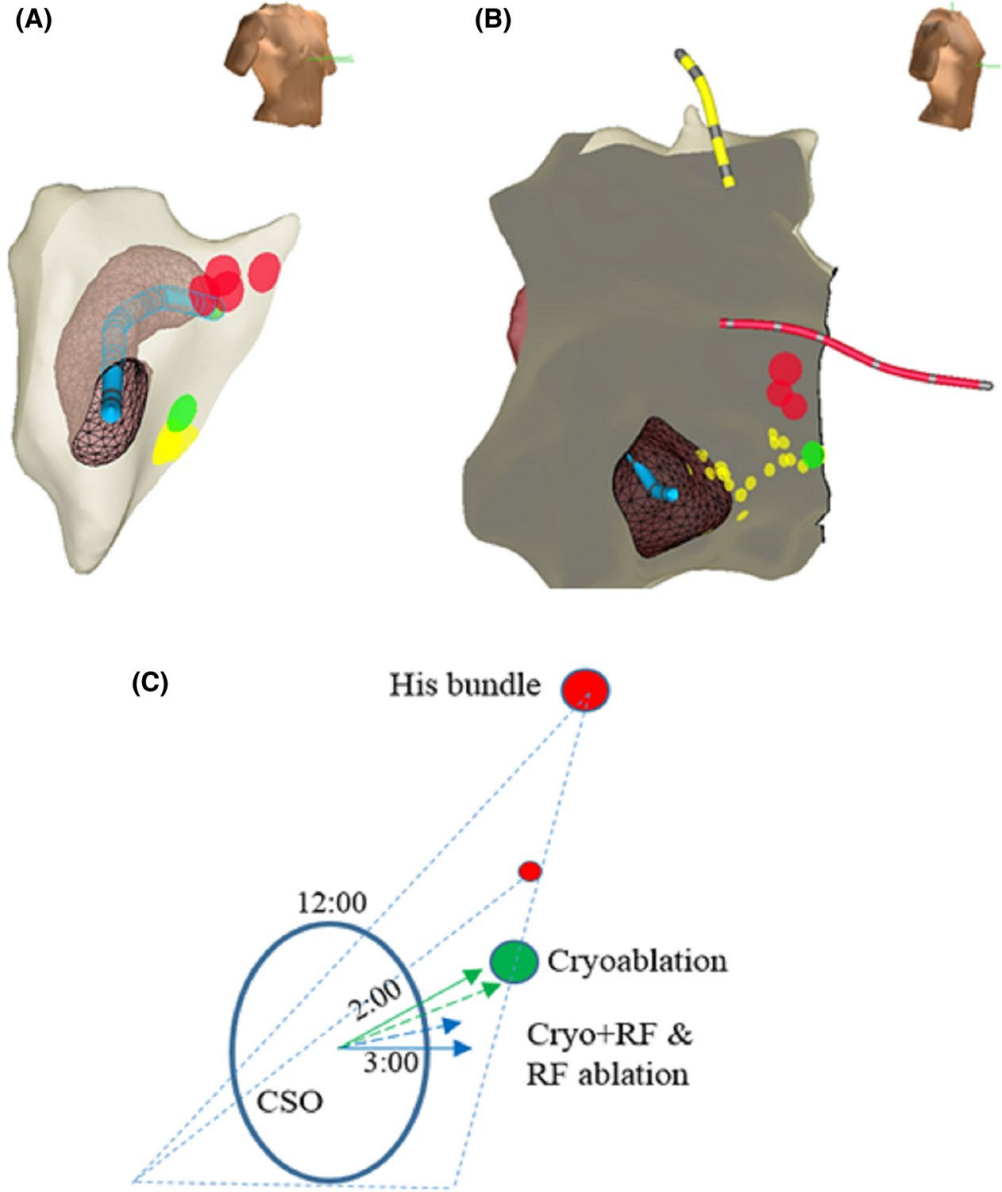
Assessment of ablation location in different KT geometry. A, A case with a wide KT (ie, the lowest recorded HB (red dots) was high above the CSO roof). The RF ablation success site (green dot) was at the CSO 3:00 o'clock direction. The vertical ablation location was at 36% of the KT height (yellow dots: insurance ablation sites). B, A case with a narrow KT (ie, the lowest recorded HB was close to the CSO roof). The ablation success site was still at around CSO 3:00 o'clock, but its vertical location was at 70% of the KT height (yellow dots: cryoablation site). C, In a wide (His depicted in a large red solid circle) and a narrow (His depicted in a small red solid circle) KTs (triangle with blue dashed lines), the median (solid arrow) and mean (dashed arrow) directional location of the cryo (green) and RF ablation (blue) success sites were depicted. While the directional location using CSO as reference was the same in either a wide or a narrow KT, the vertical location using KT height as reference was markedly different between a wide and a narrow KT anatomy. CSO, coronary sinus ostium; KT, Koch's triangle

By examining the individual cases we found that there was a caveat in describing the success ablation site by using the parameter of vertical location expressed as % of the KT height in different KT anatomy (Figure 3). Figure 3A was taken from a patient with a relatively wide KT (the lowest recorded HB was way above the CSO roof). The ablation success site was at CSO 3:00 o'clock direction, and its vertical location was at 36% of the KT height (ablation location was between the middle and lower one third of the KT). Figure 3B was taken from a patient with a relatively narrow KT (ie, the lowest recorded HB was close to the CSO roof), with ablation success site directional location still at CSO 3:00 o'clock, the vertical location became at 70% of the KT (ie, between the upper and middle third of the KT).

Transient Mobitz 1 second degree AV block occurred in 4 (15%) of Group 1 patients during or immediately after stopping cryoablation, and all recovered at the end of the procedure. Two (29%) patients in Group 2 had Mobitz 1 second degree AV block in their overnight telemetry monitoring, which disappeared in their follow‐up studies. In the entire cohort there was no patient who developed irreversible heart block. Three patients were lost to follow‐up. During a median follow‐up of 12 month (n = 49), three patients with cryoablation complained of palpitations without documented tachycardia, two of them had isolated premature ventricular contractions in their follow‐up event monitors. Only one patient with RF ablation only in Group 2 (who had a postablation residual retrograde dual AVN pathway without antegrade AH jump or inducible AV nodal echo beat) had a documented tachycardia recurrence (2.1%).

## 4 DISCUSSION

With 3D electroanatomic mapping, we can create an individual KT in each case and use each patient's reconstructed CSO as the reference to precisely define the ablation success sites. In this study we documented the success ablation site in cryoablation was mostly (median) around the CSO 2:00 o'clock direction, and in RF ablation around its 3:00 o'clock direction, which was significantly lower than that of cryoablation success site. Although this result is unsurprising for those who practice both methods of ablation of AVNRT, this is first study that clearly delineated the success ablation sites of cryoablation alone or any procedure including RF ablation.

In addition, we found that the success vertical ablation location using the KT height as the reference in cryoablation is also significantly higher than that of RF ablation. However, because of the wide variation of the geometry of the KT, using this parameter can be misleading. This is illustrated in Figure 3C, in which both a wide (HB is depicted in a large red solid circle) and a narrow (His depicted in a small red solid circle) KTs are illustrated. While describing ablation success site by using the CSO as reference the directional location is not affected by the degree of “wideness” of the KT, the vertical location using the KT height as reference is markedly affected by different KT anatomy.

Because cryoablation is safer compared to RF ablation, in this study we chose cryoablation as our primary energy source for SP ablation and used RF energy as rescue, except in patients deemed not suitable for cryoablation. We could achieve cryoablation success in 80% of the patients and in the rest we used additional RF ablation to achieve final success. Our overall acute ablation success rate was 100%, with 2.1% recurrence at 1‐year follow‐up, without the complication of long‐term irreversible heart block. Similar to our results, with combined cryo and RF ablation approach, Miyamoto reported that in 14 adult patients with AVNRT 79% achieved direct cryoablation success, and 14% required additional RF ablation to achieve final success, with one failed ablation.^10^

By using cryothermal energy only for AVNRT ablation, in a multicenter retrospective study in Japan, Okishige et al reported an overall acute success rate of 99.3% and recurrence rate at 1 year of 3.9%.^11^ In 274 children diagnosed as AVNRT, Karacan et al reported a 100% acute success rate and a 4.4% long‐term recurrence rate. However, they used a larger tip (8 mm) cryocatheter in 36% of their patients, and delivered a longer initial cryolesion (6 minutes), and more additional 4‐minutes lesions (≥6; to form a linear lesion) than our cryoablation cases.^12^

The fact that the number of unsuccessful cryoablation attempts in Group 1 who had cryoablation success was significantly higher than the number of unsuccessful RF ablation attempts in Group 2 patients, and, when the attempted cryoablation at higher KT failed (at CSO 2:00 o'clock), the rescue RF ablation could still be successful at lower CS ostium (3:00 o'clock direction, same direction as the patients with only RF ablation) attests the better efficacy of RF ablation compared to that of cryoablation.^13^ We encountered no short‐ or long‐term complication of using this “ice and fire” approach. Therefore, we suggests that it is reasonable to make an earlier switch from cryothermal to RF energy if the initial cryoablation is deemed ineffective, and start RF ablation targeting at the CS ostium 3:00 o'clock site.

Since there is no good marker for SP, clinicians are always striving to find a better way to ablate the slow pathway. In 1992, Hassaigairre et al and Jackman et al independently reported that a low‐amplitude activity with a slow rate of rise between atrial and ventricular electrograms, or atrial electrograms with a low‐amplitude and low‐frequency component followed by a high‐amplitude and high‐frequency component (ie, slow pathway potentials) could be helpful in guiding AVNRT ablation.^6^, ^7^ In a randomized study, Efremidis et al found that the efficacy and safety of SP ablation were similar using either potential‐guided or anatomic‐guided approach.^14^ We used anatomical‐guided approach and did not actively seek SP potential in our mapping‐ablation procedure.

Several reports showed that by using 3D electroanatomic mapping and a superimposed atrial electrogram voltage map, a low‐voltage bridge in the KT can be visualized and used as a surrogate for the slow pathway ablation, and increase ablation accuracy and reduce recurrence rates.^15^, ^16^, ^17^, ^18^ Drago et al reported that by using voltage mapping of KT, combined with the search for the slow potential signal in 'low‐voltage bridges' to guide cryoablation of AVNRT in children was very effective (with 100% success) in guiding cryoablation of AVNRT in pediatric patients.^19^ We were not doing voltage mapping during this study period. Anyway, our findings in this study, using CS ostium as reference in SP ablation, for cryoablation success site at 2:00 o'clock and for RF ablation at 3:00 o'clock, will add another useful tool in guiding the ablation target. Although we speculate that our method can be applied to adults and to patients with congenital heart disease or having persistent left superior vena cava, since all our patients had structurally normal heart, this will await future studies to confirm.

One obvious limitation in this retrospective research is that since the operator is comfortable in using cryoablation starting at higher site in the KT because of its safety feature, if success was achieved at CS ostium 2 o'clock direction without first attempting cryoablation at 3:00 o'clock, we would not know if lower lesions might be successful as well. On the other hand, when RF ablation at CS ostium 3:00 o'clock was successful, we would not know if it can also achieve success at higher site without causing AV nodal injury.

In the 3D electroanatomic mapping the boundary of the triangle of Koch is defined by the lowest His bundle recording site, the septal tricuspid valve annulus, and the posterior margin of the ostium of CS as a surrogate of Tendon of Todaro. Of these, the lowest HB recording site may not be well mapped for each patient. However, this should not affect our results since we use the CS ostium as our reference point. Besides, the reconstructed shape of the CS ostium was not by any stretch of imagination precise even by advancing, withdrawing, and deflecting of the shaft section of the decapolar catheter as described in our methods. Anyway, the CS ostium is important only as the directional reference for a successful ablation in the clinical setting, not its precise shape.

## 5 CONCLUSIONS

In this retrospective study we documented the success ablation site in cryoablation was mostly around the CS ostium 2:00 o'clock direction, and in RF ablation around its 3:00 o'clock direction. Our findings will add another useful tool in guiding the ablation target. Future prospective studies will be needed in confirming this method's efficacy.

## CONFLICT OF INTEREST

Authors declare no conflict of interests for this article.

## DISCLOSURES

The protocol for this research project has been approved by Memorial Healthcare System's institutional review board (Approval No. MHS2018.024; Date of Approval, 06/12/2019) and it conforms to the provisions of the Declaration of Helsinki.

## References

[1] Wang P , Cheng Z . How to perform radiofrequency and cryoablation for AV nodal reentrant tachycardia In: Al‐Ahmad A, Callans D, Hsia H, Natale A, Oseroff O, Wang P, editors. Hands‐On Ablation: The Expert’s Approach. Minneapolis, MN: Cardiotext Publishing; 2013 p. 51–58.

[2] Sumitomo N , Tateno S , Nakamura Y , Ushinohama H , Taniguchi K , Ichikawa R , et al. Clinical importance of Koch's triangle size in children: a study using 3‐dimensional electroanatomical mapping. Circ J. 2007;71:1918–21. 18037746 10.1253/circj.71.1918

[3] Goldberg CS , Caplan MJ , Heidelberger KP , Dick M II . The dimensions of the triangle of Koch in children. Am J Cardiol. 1999;83:117–20. 10073798 10.1016/s0002-9149(98)00794-2

[4] Yamaguchi T , Tsuchiya T , Nagamoto Y , Miyamoto K , Sadamatsu K , Tanioka Y , et al. Anatomical and electrophysiological variations of Koch's triangle and the impact on the slow pathway ablation in patients with atrioventricular nodal reentrant tachycardia: a study using 3D mapping. J Interv Card Electrophysiol. 2013;37:111–20. 23408048 10.1007/s10840-012-9769-z

[5] Van hare GF , Javitz H , Carmelli D , Saul JP , Tanel RE , Fischbach PS , et al. Prospective assessment after pediatric cardiac ablation: demographics, medical profiles, and initial outcomes. J Cardiovasc Electrophysiol. 2004;1597:759–70. 10.1046/j.1540-8167.2004.03645.x 15250858

[6] Haissaguerre M , Gaita F , Fischer B , Commenges D , Montserrat P , d'Ivernois C , et al. Elimination of atrioventricular nodal reentrant tachycardia using discrete slow potentials to guide application of radiofrequency energy. Circulation. 1992;85:2162–75. 1591833 10.1161/01.cir.85.6.2162

[7] Jackman WM , Beckman KJ , McClelland JH , Wang X , Friday KJ , Roman CA , et al. Treatment of supraventricular tachycardia due to atrioventricular nodal reentry by radiofrequency catheter ablation of slow‐pathway conduction. N Engl J Med. 1992;327:313–8. 1620170 10.1056/NEJM199207303270504

[8] Seslar SP , Kugler J , Batra AS , Collins KK , Crosson J , Dubin AM , et al. The Multicenter Pediatric and Adult Congenital EP Quality (MAP‐IT) Initiative‐rationale and design: report from the pediatric and congenital electrophysiology society's MAP‐IT taskforce. Congenit Heart Dis. 2013;8:381–92. 23663492 10.1111/chd.12084

[9] Harris PA , Taylor R , Thielke R , Payne J , Gonzalez N , Conde JG . Research electronic data capture (REDCap) – a metadata‐driven methodology and workflow process for providing translational research informatics support. J Biomed Inform. 2009;42:377–81. 18929686 10.1016/j.jbi.2008.08.010 PMC2700030

[10] Miyamoto K , Kapa S , Mulpuru SK , Deshmukh AJ , Asirvatham SJ , Munger TM , et al. Outcome of combined cryo‐ and radiofrequency‐catheter ablation in patients with supraventricular tachycardias. J Cardiovasc Electrophysiol. 2019;1960–1966. 10.1111/jce.14068 31310387

[11] Okishige K , Okumura K , Tsurugi T , Yotsukura A , Nanbu T , Sugiura H , et al. Japan ablation registry: cryoablation in atrioventricular nodal reentrant tachycardia (“JARCANRET study”): results from large multicenter retrospective investigation. J Interv Card Electrophysiol. 2019 10.1007/s10840-019-00585-0 31367961

[12] Karacan M , Çelik N , Akdeniz C , Tuzcu V . Long‐term outcomes following cryoablation of atrioventricular nodal reentrant tachycardia in children. Pacing Clin Electrophysiol. 2018;41(3):255–60. 29318633 10.1111/pace.13277

[13] Opel A , Murray S , Kamath N , Dhinoja M , Abrams D , Sporton S , et al. Cryoablation versus radiofrequency ablation for treatment of atrioventricular nodal reentrant tachycardia: cryoablation with 6‐mm‐tip catheters is still less effective than radiofrequency ablation. Heart Rhythm. 2010;7:340–3. 20185106 10.1016/j.hrthm.2009.11.029

[14] Efremidis M , Sideris A , Letsas KP , Alexanian IP , Pappas LK , Mihas CC , et al. Potential‐guided versus anatomic‐guided approach for slow pathway ablation of the common type atrioventricular nodal reentry tachycardia: a randomized study. Acta Cardiol. 2009;64:477–83. 19725440 10.2143/AC.64.4.2041612

[15] Bailin SJ , Korthas MA , Weers NJ , Hoffman CJ . Direct visualization of the slow pathway using voltage gradient mapping: a novel approach for successful ablation of atrioventricular nodal reentry tachycardia. Europace. 2011;13:1188–94. 21508003 10.1093/europace/eur112

[16] Malloy L , Law IH , Von Bergen NH . Voltage mapping for slow‐pathway visualizaton and ablation of atrioventricular nodal reentry tachycardia in pediatric and young adult patietns. Pediatr Cardiol. 2014;35:103–7. 23872907 10.1007/s00246-013-0748-7

[17] Bearl DW , Mill L , Kugler JD , Prusmack JL , Erickson CC . Visualization of atrioventricular nodal reentry tachycardia slow pathways using voltage mapping for pediatric catheter ablation. Congenit Heart Dis. 2015;10:E172–179. 25682958 10.1111/chd.12252

[18] Reddy CD , Ceresnak SR , Motonaga KS , Avasarala K , Feller C , Trela A , et al. Bridge to success: a better method of cryoablation for atrioventricular nodal reentrant tachycardia in children. Heart Rhythm. 2017;14:1649–54. 28716699 10.1016/j.hrthm.2017.07.018

[19] Drago F , Battipaglia I , Russo MS , Remoli R , Pazzano V , Grifoni G , et al. Voltage gradient mapping and electrophysiologically guided cryoablation in children with AVNRT. Europace. 2018;20:665–72. 28407062 10.1093/europace/eux021

